# Effects of the pleiotropic regulator DasR on lincomycin production in *Streptomyces lincolnensis*

**DOI:** 10.1007/s00253-024-13201-7

**Published:** 2024-06-15

**Authors:** Huihui Pai, Yiying Liu, Chuanbo Zhang, Jianyu Su, Wenyu Lu

**Affiliations:** 1 School of Chemical Engineering and Technology, Tianjin University, Tianjin, PR China; 2 Frontiers Science Center for Synthetic Biology, Tianjin University, Tianjin, PR China; 3 Key Laboratory of System Bioengineering (Tianjin University), Ministry of Education, Tianjin, PR China; 4 Key Laboratory of the Ministry of Education for Conservation and Utilization of Special Biological Resources in the Western, Yinchuan, 750021 China; 5 College of Life Science, Ningxia University, Yinchuan, 750021 Ningxia China

**Keywords:** DasR, Lincomycin, *S. lincolnensis*, Transcriptional regulators, Transcriptome analysis

## Abstract

**Abstract:**

The lincoamide antibiotic lincomycin, derived from *Streptomyces lincolnensis*, is widely used for the treatment of infections caused by gram-positive bacteria. As a common global regulatory factor of GntR family, DasR usually exists as a regulatory factor that negatively regulates antibiotic synthesis in *Streptomyces*. However, the regulatory effect of DasR on lincomycin biosynthesis in *S. lincolnensis* has not been thoroughly investigated. The present study demonstrates that DasR functions as a positive regulator of lincomycin biosynthesis in *S. lincolnensis*, and its overexpression strain *OdasR* exhibits a remarkable 7.97-fold increase in lincomycin production compared to the wild-type strain. The effects of DasR overexpression could be attenuated by the addition of GlcNAc in the medium in *S. lincolnensis*. Combined with transcriptome sequencing and RT-qPCR results, it was found that most structural genes in GlcNAc metabolism and central carbon metabolism were up-regulated, but the lincomycin biosynthetic gene cluster (*lmb*) were down-regulated after *dasR* knock-out. However, DasR binding were detected with the DasR responsive elements (*dre*) of genes involved in GlcNAc metabolism pathway through electrophoretic mobility shift assay, while they were not observed in the *lmb*. These findings will provide novel insights for the genetic manipulation of *S. lincolnensis* to enhance lincomycin production.

**Key points:**

• *DasR is a positive regulator that promotes lincomycin synthesis and does not affect spore production*

• *DasR promotes lincomycin production through indirect regulation*

• *DasR correlates with nutrient perception in S. lincolnensis*

**Supplementary Information:**

The online version contains supplementary material available at 10.1007/s00253-024-13201-7.

## Introduction

Lincomycin is an important lincoamide antibiotic produced by *S. lincolnensis*. It is commonly used in the clinical treatment of infections caused by gram-positive bacteria, such as respiratory tract, urinary system, skin and soft tissue infections and other diseases, such as otitis media and eye diseases (Zhang et al. 1992). In the past decades of production practice, the industrial strains of lincomycin are mainly mutated by physical or chemical methods. However, these approaches are confronted with challenges of high reaction demands and prolonged reaction times. Therefore, there is an urgent need for a genetically engineered strain capable of producing lincomycin via fermentation to meet the demands of industrial production. The biosynthesis process of lincomycin has been extensively studied and its synthetic pathway has become increasingly clear. The widely recognized synthesis of lincomycin can be divided into three parts (Neusser et al. 1998; Spizek and Rezanka 2017; Wang et al. 2020): the synthesis of Methylthiolincosamide (MTL), the synthesis of 4-propyl-*L*-proline (PPL), and the subsequent condensation and modification processes. However, the current understanding of the regulatory mechanism underlying lincomycin biosynthesis in *S. lincolnensis* remains limited, impeding its potential for industrial production.

DasR belongs to the GntR family of transcriptional suppressor proteins. As a global transcriptional regulatory protein, DasR binds to the DasR responsive element (*dre*) in the gene promoter region of *Streptomyces* species to exert transcriptional regulation (Colson et al. 2007; Rigali et al. 2008). These binding sites are widely distributed, covering genes related to a number of primary and secondary metabolisms such as phosphotransferase system (PTS), aerial mycelium growth, chitin degradation, antibiotic synthesis, etc. (Colson et al. 2007; Nazari et al. 2013; Rigali et al. 2006), suggesting an important global regulatory role played by DasR. *dasR*-deficient *S. griseus* showed abnormalities in both aerial mycelial growth and spore production when glucose was used as a carbon source. In *S. griseus*, the gene *dasA* has been identified alongside the gene *dasR*, which is located adjacent to it but transcribed in the opposite direction. Mutation or overexpression of both genes has been reported to affect the morphology of *S. griseus* strains (Seo et al. 2002).

DasR also has an important regulatory role in antibiotic synthesis. In most cases, DasR exerts a negative regulatory effect on antibiotic synthesis (Huang et al. 2022). In the *dasR* deficient *S. coelicolor* BAP29, actinomycetin production was significantly increased (Rigali et al. 2004). In *S. verticillus*, though there is no *dre* sequence in the bleomycin biosynthesis gene cluster, DasR exerts an inhibitory effect on the production of bleomycin through indirect regulation (Chen et al. 2020).

*Streptomyces*, as a microorganism widely living in soil, usually takes the hydrolysates of chitin as its food source. Chitin is digested into N-acetylglucosamine (GlcNAc) and dimer (GlcNAc)_2_ and released into the environment to be re-taken by new cells (Colson et al. 2008). GlcNAc is an important signaling molecule (Rigali et al. 2006), which is transferred to the intracellular by phosphotransferase system and then phosphorylated to generate GlcNAc-6P, and then deacetylated by NagA to generate GlcN-6P (Nothaft et al. 2010). Both GlcNAc-6P and GlcN-6P specifically inhibit the binding of DasR to the promoter of the gene, releasing the original DNA target, which is an important effector molecule for DasR regulation (Rigali et al. 2006), and then catalyzed by NagB to generate Fru-6P, which can enter the glycolytic pathway (Chen et al. 2019). DasR binding sites were also generally found in the existing upstream of many GlcNAc-related genes, which are the targets of DasR, including the genes of the chitinolytic system, as well as the transport and metabolism genes of GlcNAc and its polymers. The activity of DasR and its response to GlcN-6P and GlcNAc-6P levels depend on environmental conditions: adding high concentrations of GlcNAc in nutrient-poor conditions (e.g., on minimal medium) enhances antibiotic production; however, GlcNAc inhibited antibiotic production in enriched medium (Van Wezel et al. 2009). These results indicate that the regulation of DasR-GlcNAc is influenced by the nutrient level of bacteria.

The structural genes, resistance genes, and regulatory genes within the lincomycin biosynthetic gene cluster have been relatively well studied. The findings suggest that the regulatory role of DasR in *Streptomyces* growth, differentiation, and antibiotic production is intricate and significant. However, the regulatory mechanism of global factor DasR on the biosynthesis of lincomycin by *S. lincolnensis* remains elusive. The compound lincomycin serves as the prototypical member of the lincoamide antibiotics and elucidating the role of DasR on regulating lincomycin production in *S. lincolnensis* holds significant implications for enhancing the synthesis of lincoamide antibiotics. Therefore, we investigated the regulatory mechanism of the global factor DasR on the biosynthesis of lincomycin in *S. lincolnensis* by engineering bacterial construction and fermentation, transcriptome sequencing, and in vitro binding assay of DasR protein and gene.

In this study, the wild-type *S. lincolnensis* NBRC_13054 was used as the original starting strain to study the regulation effect of DasR on lincomycin biosynthesis. DasR is proved to be a positive regulator for lincomycin biosynthesis, and the effects of DasR on regulating lincomycin production in *S. lincolnensis* were analyzed by transcriptome sequencing, RT-qPCR and electrophoretic mobility shift assay. It was discovered that DasR potentially exerts a global impact by directly regulating the carbon metabolism, thereby influencing lincomycin production.

## Materials and methods

### Bacteria, plasmids, and media

All plasmids and strains used or constructed in this study are listed in Table 1. *Escherichia coli* strains were cultivated in LB (Luria-Bertani) medium at 37 °C. *S. lincolnesis* strains were cultured on MS (mannitol soya flour) medium at 28 °C for spore preparation and phenotypic observation, in YEME (yeast extract-malt extract) medium at 28 °C for cellular growth, or in ISP-2 media at 28 °C for lincomycin fermentation.

**Table 1:** Bacterial strains and plasmids used in this study

Strains or plasmids	Descriptions	Source or reference
*S. lincolnensis* strains		
NBRC_13054	Wild-type, lincomycin producer	(Lin et al. 2012)
*DdasR*	NBRC_13054 with in-frame deletion of *dasR*	This work
*OdasR*	NBRC_13054 ɸC31 attB::pIB139-*dasR*	This work
*E. coli* strains		
*E. coli* DH5α	Routine cloning host	TransGen Biotech
*E. coli* BL21 (DE3)	Protein heterologous expression host	TransGen Biotech
ET12567 (pUZ8002)	dam-13::Tn9 dcm-6 hsdM; containing the nontransmissible RP4, derivative plasmid pUZ8002	(Wang et al. 2014)
Plasmids		
pKCcas9dO	A CRISPR/Cas9 editing plasmid harboring an actII-orf4-specifc gRNA and two homologous arms for in-frame deletion, aac(3)IV, pSG5	(Huang et al. 2015)
pIB139	Integrative vector based on ɸC31 int/attP, aac(3)IV, oriT RK2, PermE*	(Wilkinson et al. 2002)
pCold II	CSPA promoter, His-tag, *kan*	Novagen
pKCcas9dO-*dasR*	A CRISPR/Cas9 editing plasmid harboring a *dasR*-specifc gRNA and two homologous arms for in-frame deletion, aac(3)IV, pSG5	This work
pIB139-*dasR*	pIB139 harboring *dasR* under control of PermE*	This work
pCold II-*dasR*	pCold II-derived plasmid carrying *dasR* for protein expression and purification	This work

### Strain construction

A CRISPR/Cas9-mediated genetic editing method (Huang et al. 2015) was used for the deletion of *dasR* from *S. lincolnensis* NBRC_13054 (wild type, WT). The construction process is shown in Fig. S1a. The recognition site GGG of *S. lincolnensis dasR* gene and its first 20 bp guide sequence GTCAGCAGTGCGGAGAACGA were selected through the website (https://zlab.squarespace.com/guide-design-resourcesforecast). Then, PCR amplification was performed to obtain the guide sequence and homologous arms of *dasR* gene with an upstream and downstream length of 1000 bp, using pKCcas9dO plasmid and *S. lincolnensis* NBRC_13054 genome as templates respectively. A recombinant fragment of about 2100 bp in length was obtained by fusion PCR (Fig. S1). Then, the recombinant fragment was ligated between to the *Spe*I and *Hin*dIII sites of pKCcas9dO plasmid through seamless cloning resulting in pKCcas9dO-*dasR*. The primers used in this experiment are shown in Table S1. The pKCcas9dO-*dasR* plasmid was transformed into *E. coli* ET12567 (pUZ8002), and conjugative transfer experiments were carried out according to the method reported (Hou et al. 2018). The correct clone, namely *DdasR*, was confirmed through PCR and sequencing analysis as depicted in Fig. S1b, c.

The CDS region of *dasR* gene was amplified by PCR using the genome of *S. lincolnensis* NBRC_13054 as templates and was then inserted to the *Nde*I and *Eco*RI sites of pIB139 by the same seamless cloning method resulting in pIB139-*dasR*. The primers used in this experiment are shown in Table S2. The plasmid was conjugated and transferred to *S. lincolnensis* NBRC_13054, getting strain *OdasR*. The construction process is shown in Fig. S2.

### Fermentation and lincomycin bioassay detection

*S. lincolnensis* NBRC_13054 and its derivatives were coated on spore-producing solid medium at 28 °C for 5–7 days, then 1–2 cm^2^ fresh *S. lincolnensis* moss were shoveled from the solid medium with a sterile cell spatula, inoculated into 25 mL of Fermentation Primary Medium (250 mL shaker), and fermented in the shaker (220 rpm) for 2 days at 28 °C. According to the 10% inoculum volume, the bacterial sap was transferred to 25 mL of fermentation secondary medium (250 mL shaker) and incubated at 28 °C, 220 rpm for 7 days.

One milliliter of fermentation broth at the end of fermentation was taken, and the supernatant was centrifuged and detected by high-performance liquid chromatography (HPLC). Detection conditions: column: C18 (SinoChrom ODS-BP, 4.6 × 250 mm, 5 μm, Dalian Elite Analytical Instruments Co., Ltd.); mobile phase: 50 mM ammonium acetate solution: methanol = 4:6; flow rate: 0.6 mL/min; injection volume: 20 µL; detection wavelength: 210 nm.

Dry weight was used as the index of biomass detection. After shaking the fermentation broth, 4 mL of the fermentation broth was taken into an EP tube, centrifuged and washed for sedimentation, the EP tube in which the bacterial sediment was collected was dried in an electric blower drying oven with the lid open until the mass no longer changed, and the data were recorded; the dry weight was recorded.

### RNA-Seq transcriptomic analysis

The initial strain NBRC_13054 and the knockout strain *DdasR* were selected for subsequent transcriptome sequencing analysis on days 2, 4, and 7 of fermentation, which represent the rapid growth stage of the bacterium in the pre-fermentation period, the stabilization stage in the mid-fermentation period, and the end of fermentation, respectively. Transcriptome sequencing was based on the Illumina sequencing platform, and gene expression was calculated using the FPKM (Fragments Per Kilo bases per Million reads) (Mortazavi et al. 2008) method; and NBRC_13054 (GeneBank ID: CP016438.1) was used as the control group, which was screened according to the significance of difference criterion, i.e., the differential gene expression change was more than 2-fold (|log_2_(Fold Change)|>1) and *q*-value ≤ 0.05, gene significance differential expression up- and downregulation was counted. The transcriptome raw data of WT and *DdasR* were deposited in the NCBI Sequence Read Archive (SRA) under accession no. PRJNA1077293.

### RNA extraction and quantitative real-time PCR (RT-qPCR) analyses

Total RNA was extracted using the RNAprep pure Cell/Bacteria Kit from Tengen Biotech Co., Ltd. Reverse transcription experiments were performed using the SPARK script II RT Plus Kit (With gDNA Eraser) for first-strand cDNA synthesis from Shandong Sparkjade Biotechnology Co. The enhancer 1,2,4-triazole can be added to the system optionally to improve the amplification specificity of the high GC template. The PCR primers used in the experiments are shown in Table S2, and the amplified products were best 100–150 bp long. The relative expression of the gene was calculated by 2^−ΔΔCt^.

### Construction and purification of DasR protein expression vector

The expression of soluble protein DasR by *E. coli* requires the construction of pCold II vector containing *dasR* gene. The construction process is shown in Fig. S3.

After PCR amplification of the pCold II linearized vector and *dasR* gene using primers are shown in Table S3, the target fragment was connected to the vector plasmid by seamless cloning. The insertion position of *dasR* gene was between *Ned*I and *Xho*I sites after the 6×His label. After construction, it was transformed into *E. coli* receptor state BL21 (DE3), then the correctly sequenced plasmid was stored in *E. coli* at low temperature, and the DasR expression strain containing pCold II-*dasR* vector was constructed. After determining the optimal imidazole elution concentration, the eluate containing the target protein was concentrated in a MILLIPORE protein ultrafiltration tube, and all the eluate was concentrated to about 1.5 mL.

### Protein preparation and electrophoretic mobility shift assays (EMSAs)

EMSA was performed according to the kit instructions. Probes were prepared using Cy5 fluorescent labeling for two rounds of PCR amplification. Configure 20 µL of gel migration assay system. A 6% non-denaturing polyacrylamide gel was prepared under light-avoiding conditions, electrophoresed, and the results were recorded using a fluorescent imaging system. First PCR primers for the promoter regions of the target genes are shown in Table S4.

## Result

### DasR positively regulates lincomycin production in *S. lincolnensis*

**Fig. 1:**
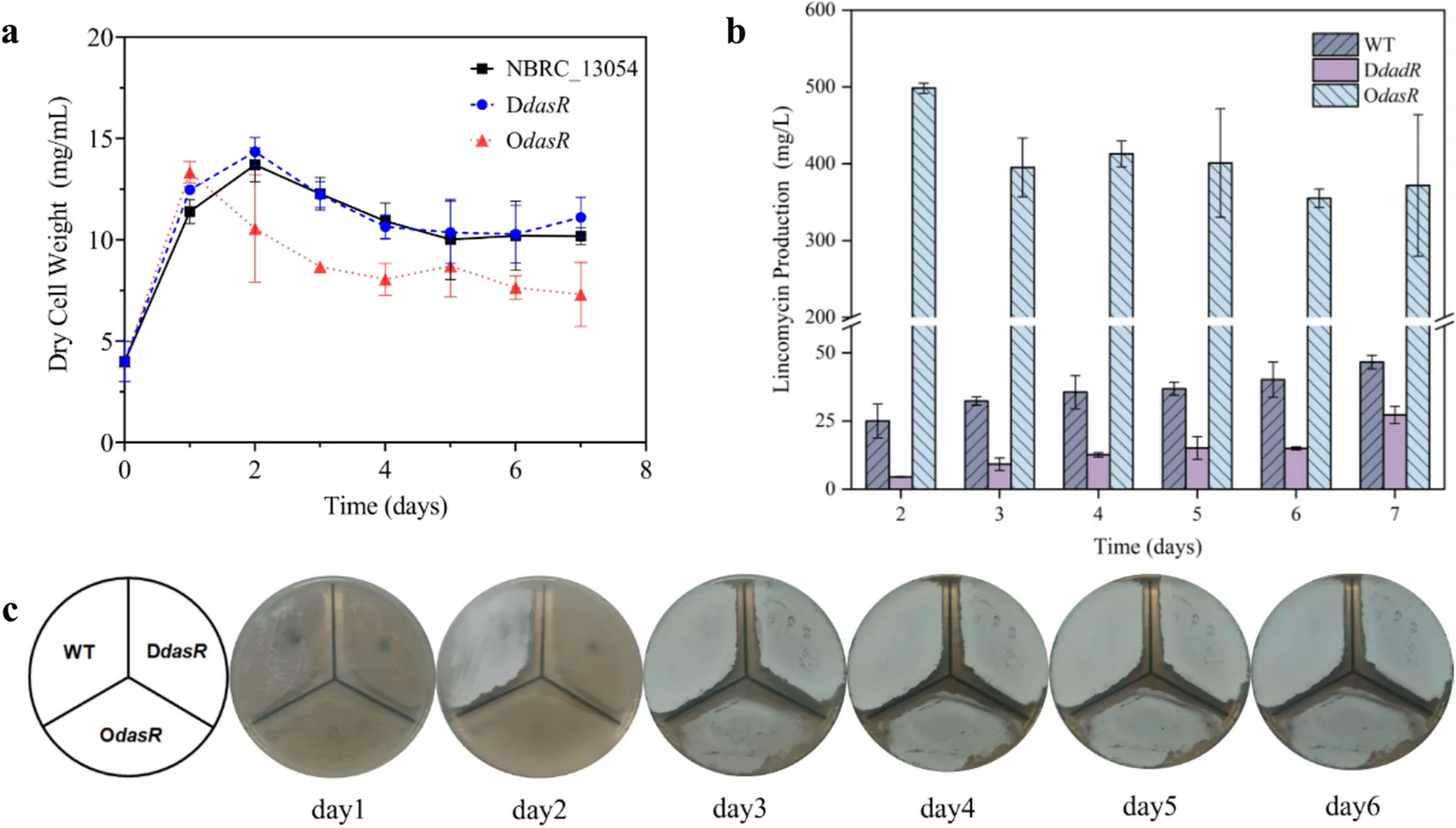
Effect of *DasR* on phenotypes of *S. lincolnesis*.  **a** Dry cell weight of strains NBRC_13054, *DdasR*, and *OdasR* in fermentation. **b** Changes in lincomycin production by WT, *DdasR*, and *OdasR* fermentation over time. **c** Morphological changes of strains WT, *DdasR*,  and *OdasR* on sporulation medium

In order to explore the effect of DasR on the lincomycin production and growth of *S. lincolnensis*, we constructed knockout (*DdasR*) and overexpression (*OdasR*) strains of the *DasR* gene. As shown in Fig. 1a, the dry cell weight (DCW) changes of *DdasR* strain and the wild-type strain (WT) are similar: both strains undergo logarithmic growth two days prior to fermentation, followed by a gradual transition to a stable stage and subsequent decline. These findings indicated that the knockout of *dasR* gene does not affect the growth of *S. lincolnensis*. However, the *OdasR* strain grew rapidly on the first day of fermentation, and its DCW dropped significantly from the second day. The overexpression of the DasR gene is speculated to enhance the transcriptional inhibitory role of DasR, thereby impacting strain growth and driving it towards a decline phase. The lincomycin production of strains in the fermentation process was significantly weakened by the deletion of the DasR gene, as depicted in Fig. 1b. Conversely, overexpression of the DasR gene led to a significant increase in lincomycin yield. However, the lincomycin titer appears to have decreased following the DCW of *OdasR*. The lincomycin titer was 27.25 mg/L, 46.63 mg/L, and 371.69 mg/L after 7 days of fermentation for WT, *DdasR*, and *OdasR* strains, respectively. Moreover, the highest lincomycin titer reached 499.86 mg/L after 2 days fermentation of *OdasR*, exhibiting a remarkable increase of 7.97 times compared to the wild-type strain. According to the results, DasR plays a positive role in lincomycin production but severely impacts the growth of *S. lincolnensis*. Therefore, phenotypic differences of the engineered strains were evaluated on the spore-producing plate (Fig. 1c). The sporulation of *DdasR* and *OdasR* strains was observed to be significantly delayed compared to that in the WT strain. Moreover, the spores produced by *OdasR* exhibited slightly light gray coloration compared to the other two strains, suggesting that DasR gene may have an effect on the morphological differentiation of *S. lincolnensis*.

### GlcNAc addition weakened the positive regulatory effect of DasR on lincomycin production

As an important signaling molecule of *Streptomyces*, GlcNAc can relieve the inhibition of global transcription factor DasR on GlcNAc metabolism, sugar transport, antibiotic synthesis, and development. To investigate its effect on lincomycin production, GlcNAc was added to the fermentation medium at final concentrations of 50 mM and 100 mM. GlcNAc was added to the fermentation medium; the production of lincomycin could not be detected for WT and *DdasR* strains. However, the addition of either 50 mM or 100 mM GlcNAc exhibited negligible impact on the production of lincomycin with *OdasR*. Therefore, 200 mM GlcNAc was then added, and the lincomycin production decreased from 371.69 to 220.00 mg/L with strain *OdasR*. These results indicate that GlcNAc or its intermediate metabolites can inhibit lincomycin production in *S. lincolnensis* under eutrophic conditions, and the inhibiting effects could be partially released by DasR.

**Fig. 2:**
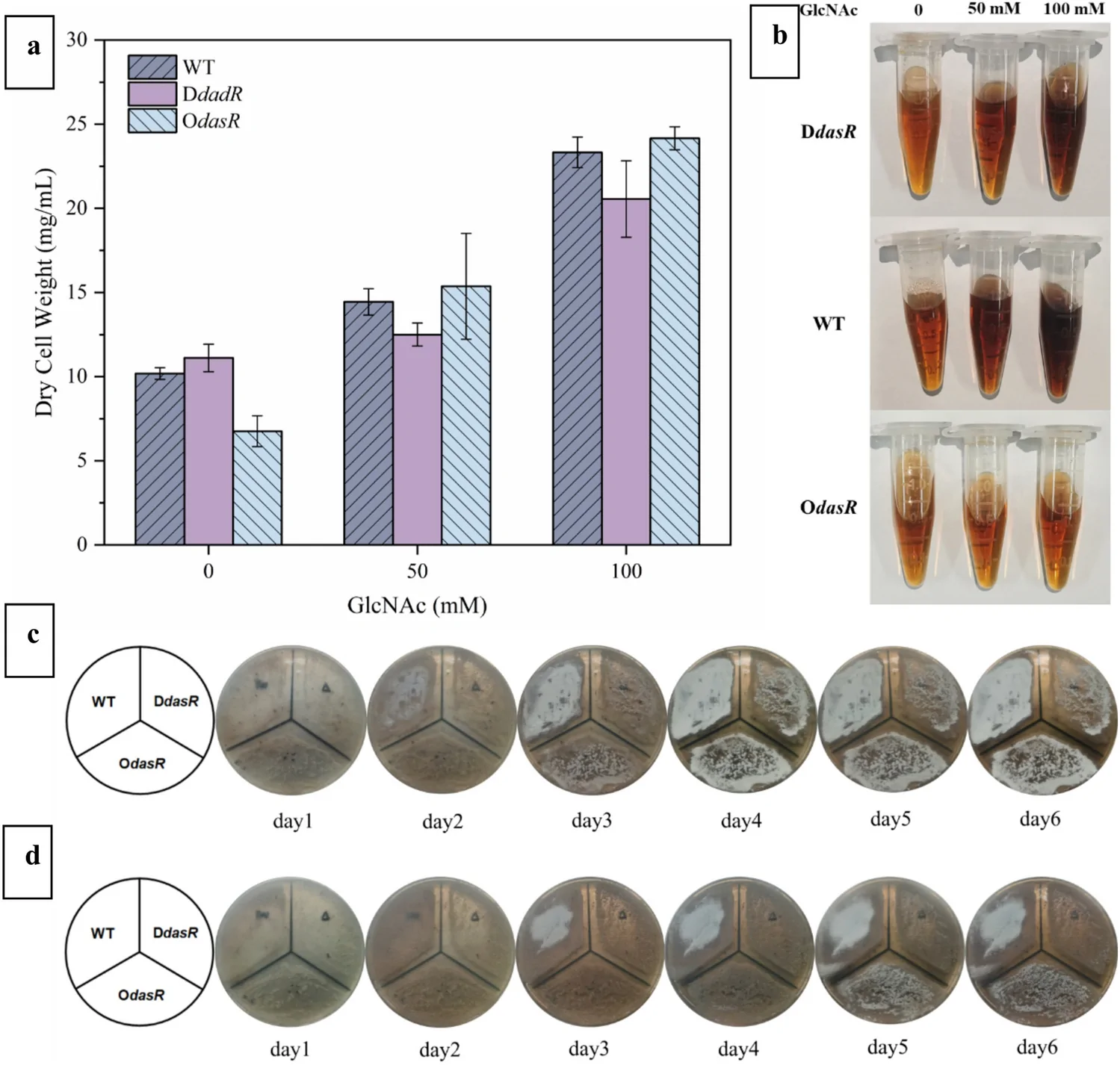
Effect of different concentrations of GlcNAc on WT, *DdasR*, and *OdasR* strains. **a** The effect of different concentrations of GlcNAc on dry cell weight of WT, *DdasR*,  and *OdasR* strains. **b** Effect of different concentrations of GlcNAc on the color of WT, *DdasR*, and *OdasR* fermentation broth. **c** Effect of 50 mM GlcNAc on sporulation of WT, *DdasR*,  and O dasR. **d** Effect of 100 mM GlcNAc on sporulation of WT, *DdasR*, and O dasR

The addition of GlcNAc also resulted in an increase in the DCW of the three strains after fermentation, as depicted in Fig. 2a since exogenous GlcNAc could provide more carbon sources for *S. lincolnensis*. These results were consistent with the increase in DCW after the addition of GlcNAc in *Streptomyces tsukubaensis* culture medium (Ordonez-Robles et al. 2018). The fermentation broth of WT and *DdasR* exhibited a deep brown color as the exogenous GlcNAc concentration increased, while the color of *OdasR* fermentation broth remained largely unchanged (Fig. 2b). This observation suggests that the regulatory network governed by DasR genes may also be involved in pigment synthesis. The addition of GlcNAc to the nutritive spore plate resulted in a significant inhibition of spore production for all three strains, particularly in the case of *DdasR* where no spores were observed on the 100 mM GlcNAc plate after 6 days of cultivation.

When GlcNAc was added for fermentation, it was also observed that the color of the fermentation broth was different from that of the fermentation broth without GlcNAc. Figure 2b shows the color comparison of the three strains. The fermentation broths of wild bacteria and *DdasR* deepened with increasing concentrations of exogenous GlcNAc, and the color of the fermentation broths of *OdasR* was essentially unchanged with the addition of GlcNAc, suggesting that the regulation between the GlcNAc and *dasR* genes may also involve the synthesis of other secondary metabolites, such as pigments.

### The transcriptome and RT-qRCR analysis of DasR-engineered strains

**Fig. 3:**
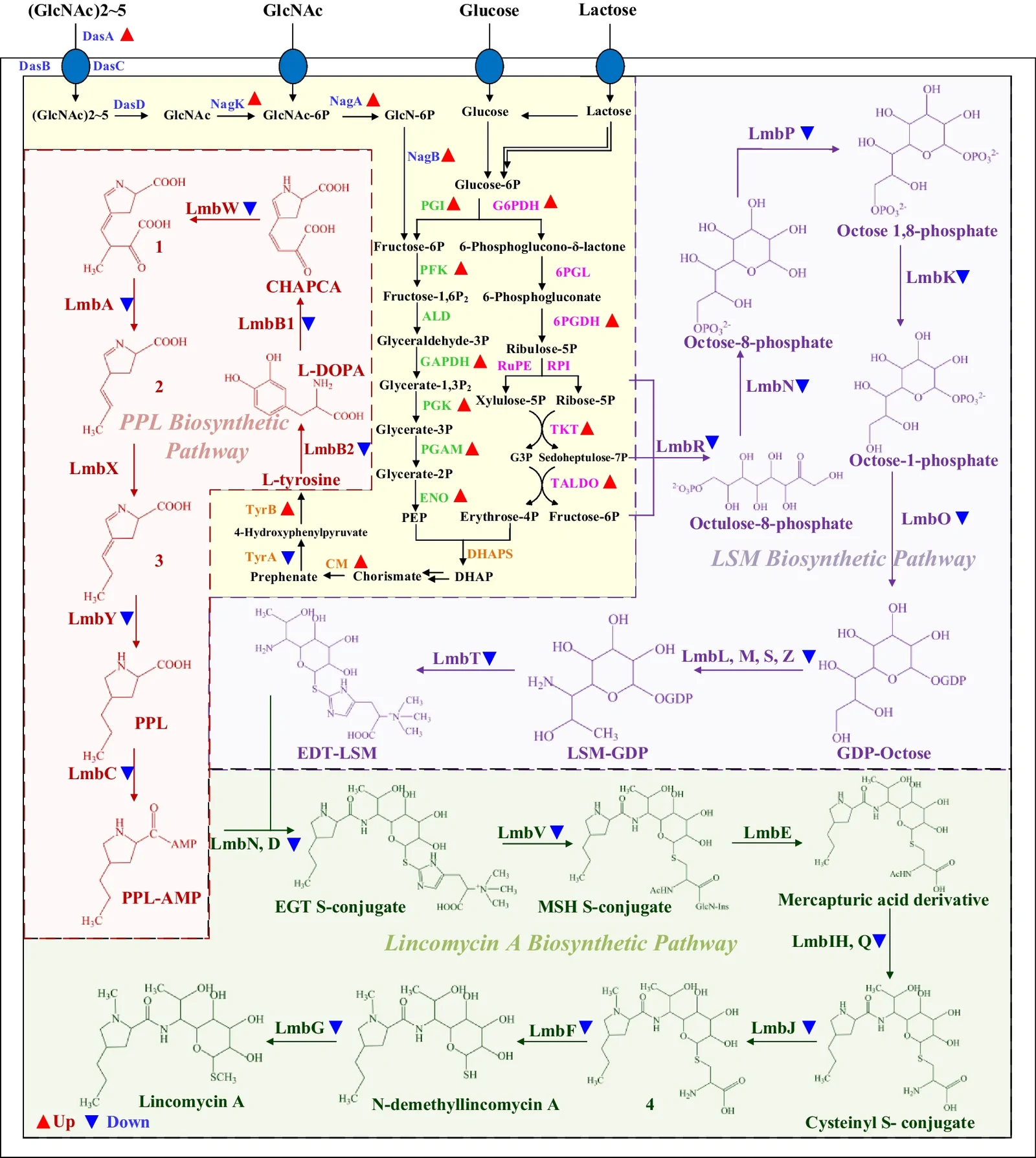
Expression of key genes in lincomycin biosynthesis in WT-48 h vs Dd-48 h

To provide a comprehensive understanding of the global regulation mediated by DasR, the transcriptome sequencing analysis was conducted on both the wild-type strain NBRC_13054 and the DasR knock-out strain *DdasR* at 48 h, 96 h, and 168 h, respectively. We focused on finding whether they contained genes within the lincomycin biosynthesis cluster and found that reduced transcript levels of cluster genes were detected in the knockout bacteria compared to the wild type in the first two periods, except on day 7. These genes covered the vast majority of structural, regulatory, and resistance genes within *lmb*, suggesting that the reduced transcript levels of the cluster genes due to the deletion of the *dasR* gene are directly responsible for the reduced lincomycin production (Fig. S4). Therefore, we generated a comprehensive map of differentially expressed genes (DEGs) associated with lincomycin biosynthesis based on the transcriptome sequencing results at 48 h, as shown in Fig. 3. After DasR gene knockout, all structural genes involved in lincomycin biosynthesis gene cluster were downregulated in the process of synthesis of LSM and PPL and their condensation. However, in the part of primary metabolism, some genes in glycolysis and pentose phosphate pathway are basically transcriptionally upregulated due to the deletion of *dasR*. Moreover, genes related to the metabolism of signaling molecules GlcNAc, such as *dasA*, *nagK*, *nagA*, and *nagB*, are also transcriptionally upregulated due to the deletion of *dasR* (Fig. S4). These results indicated that DasR may play a certain regulatory relationship with GlcNAc metabolism, central carbon metabolism, and secondary metabolism.

The purple part is the biosynthesis of LSM, the red part is the biosynthesis of PPL, the green part is the condensation of the two, and the yellow part is the basic metabolic pathway, including some key processes of glycolysis, pentose phosphate pathway, L-tyrosine synthesis of branching acids, and GlcNAc transport from extracellular to intracellular metabolism. The red triangle upward indicates that the knockout bacteria has upregulated transcription compared to the original bacteria, and the blue triangle downward indicates that the knockout bacteria has downregulated transcription compared to the original bacteria. 1 indicates 4-(3-carboxy-2-methylpropyl)-2,3-dihydro-1 H-pyrrole-2carboxylic acid. 2 indicates (E)-4-(prop-1-en-l-yl)-3,4-dihydro-2 H-pyrrole-2-carboxylic acid. 3 indicates (E)-4-propylidene-3,4-dihydro-2 H-pyrrole-2-carboxylic acid. 4 indicates S-(3,4,5-trihydroxy-6-(2-hydroxy-1-(1-methy1-4-propylpyrolidine-2-carboxamido)propyl)tetrahydro-2 H-pyran-2-yl)cysteine.

**Fig. 4:**
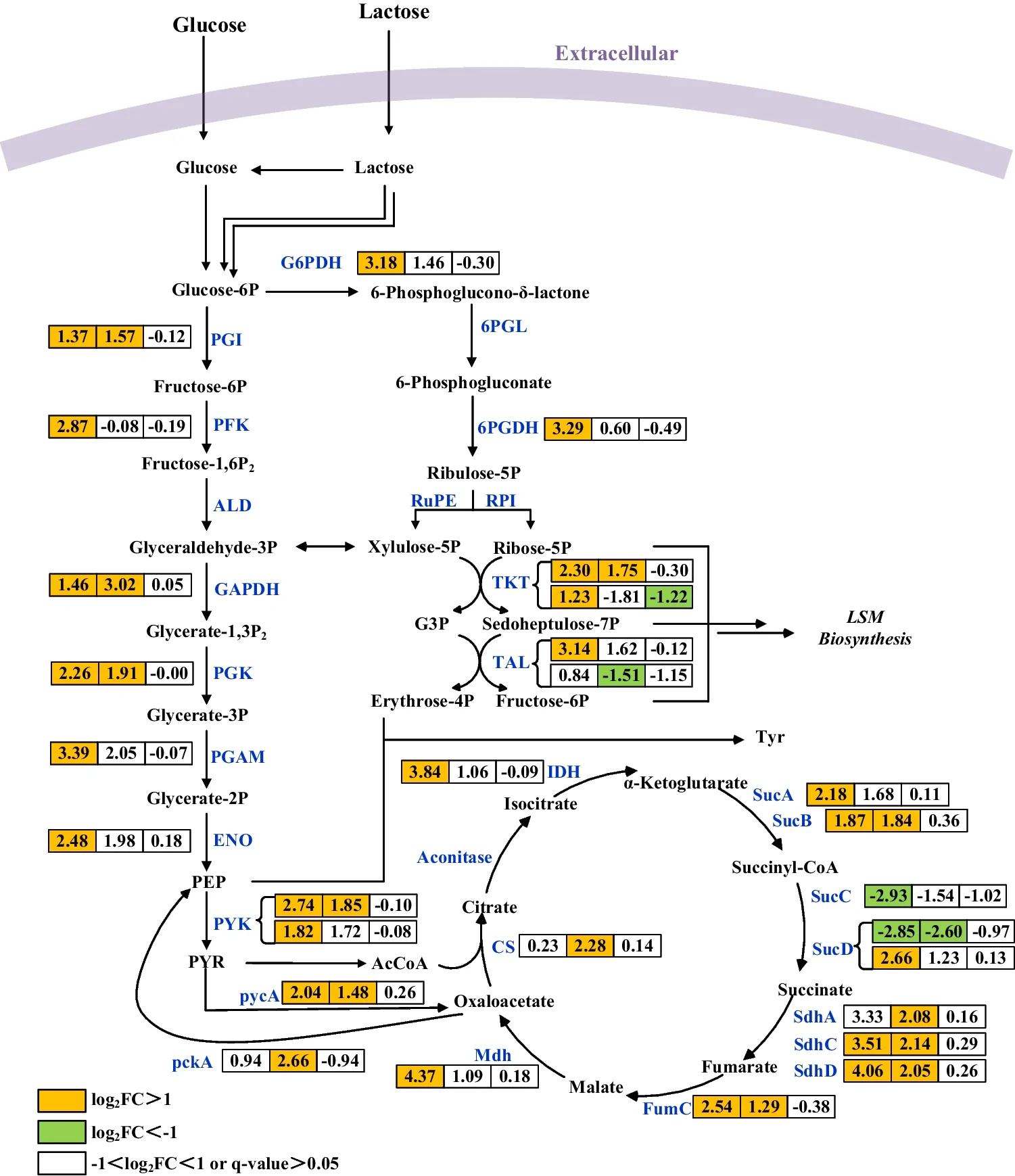
Differential expression genes related to central carbon metabolism in the WT vs Dd of *S. lincolnensis*. The three boxes next to enzymes in the metabolic pathway represent the log_2_ (Fold Change) of the gene at 48, 96, and 168 h, respectively

The DEGs related to central carbon metabolism at 48 h, 96 h, and 168 h of fermentation were then collected and exhibited in Fig. 4. The overall trend was that the transcription level of the majority of genes in central carbon metabolism of *DdasR* was significantly upregulated at 48 and 96 h, whereas there were no significant differences in other genes at 168 h except *slinc_7065*, which encodes transketolase (TKT) and showed a significant downregulation compared to the wild type (WT). These results further suggests that the regulation of central carbon metabolism by DasR is closely related to fermentation time.

**Fig. 5:**
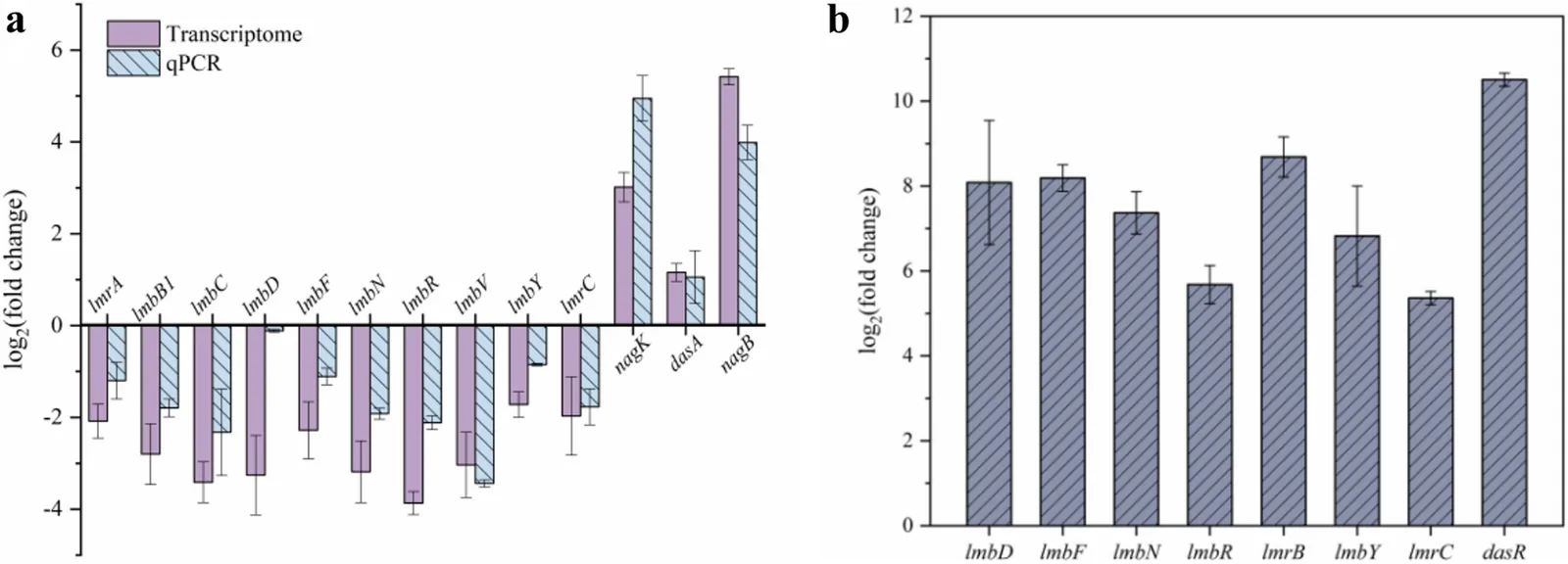
RT-qPCR analyses on genes related to lincomycin biosynthesis between strains WT, *DdasR*, and *OdasR.* **a** Comparison of transcriptome and RT-qPCR results of WT and *DdasR*. **b** RT-qPCR results of some genes on the second day of *OdasR* fermentation

In order to verify the accuracy of transcriptome sequencing, genes concerning lincomycin biosynthesis and GlcNAc metabolism were selected for RT-qPCR analysis (Fig. 5a, b). As shown in Fig. 5a, the differentially expressed genes analyzed by RT-qPCR were consistent with the transcriptome sequencing results. The transcription of *lmb* genes in *OdasR* was also analyzed by RT-qPCR (Fig. 5b), compared with WT strains; the transcription levels of structural genes and resistance genes in the *lmb* cluster are significantly improved, which explains the higher lincomycin production in *OdasR* strain.

### Analysis of DasR transcriptional regulatory targets in *S. lincolnensis *by EMSA

**Fig. 6:**
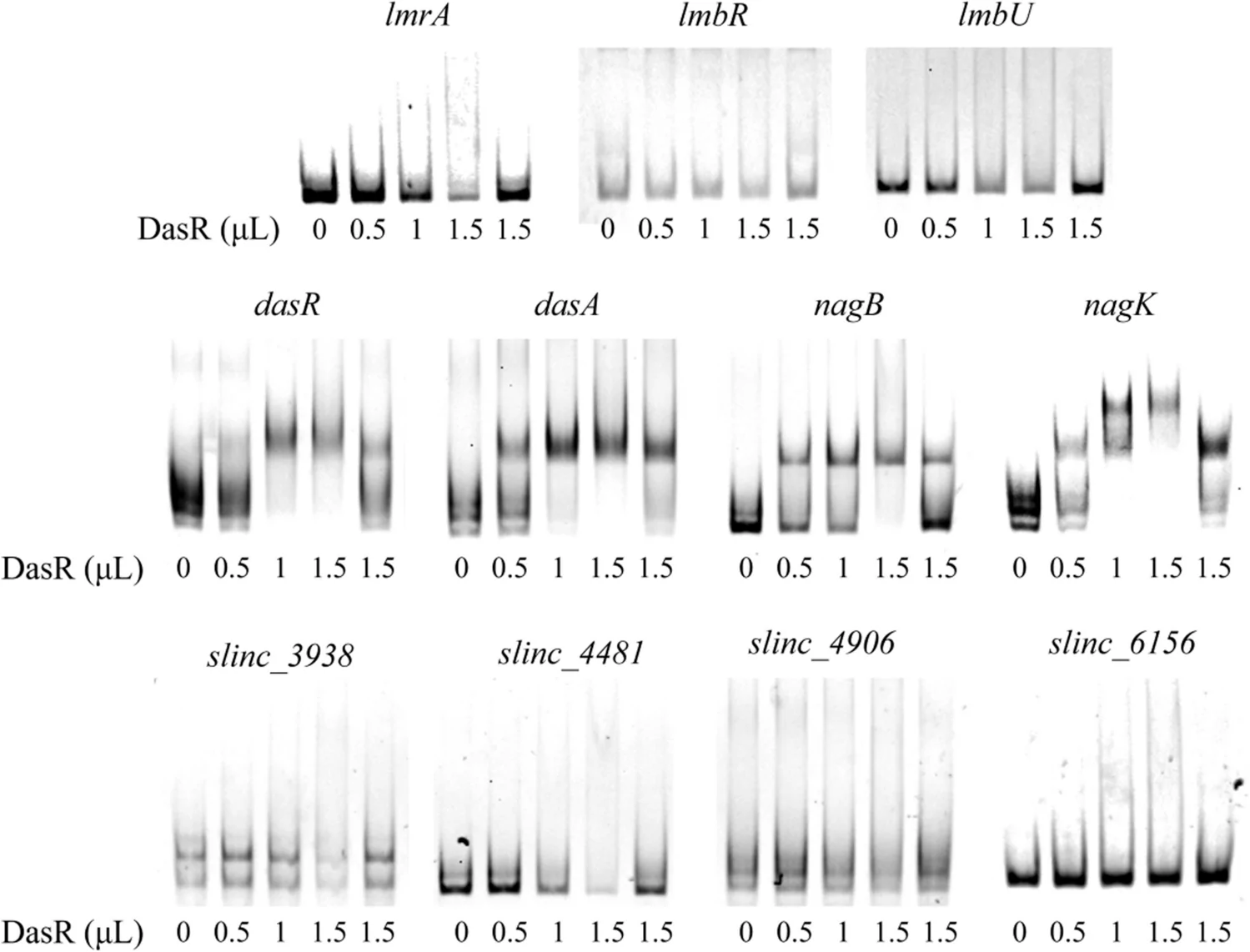
EMSAs of DasR protein and 50 ng/µL target gene probe. The fifth lane of each group in the Fig. 6 is the system with additional 10 mg/mL salmon sperm

It has been reported that DasR can specifically bind to a 16 bp sequence of gene promoter region, ASTGGTCTAGACCAST, to exert transcriptional regulation. Therefore, this sequence was uploaded to MEME/MAST (https://meme-suite.org/meme/doc/mast.html) to predict potential DasR binding sites in *S. lincolnensis* NBRC_13054. Relevant attention was directed towards the genes within the Lincomycin biosynthetic gene cluster and GlcNAc metabolism. Figure S5 shows the Weblogo of the *dre* sequence and the upstream region of genes predicted by MEME that may be bound by DasR. A total of seven binding sites were predicted, including three genes (*lmrA*, *lmbR*, and *lmbU*) located in the Lincomycin biosynthetic gene cluster, three genes (*dasA*, *nagB*, and *nagK*) in GlcNAc metabolism and *dasR* itself. Previous studies have shown that the *dasABCD* gene, involved in GlcNAc metabolism in *S. coelicolor* and *S. verticillus*, is located within the same transcriptional unit and transcribed in the opposite direction to the upstream adjacent *dasR* gene, as well as downstream adjacent *nagB*, *nagK*, and *nagA* genes present within the same transcriptional unit in their respective genomes (Chen et al. 2019). There were 90.12%, 83.52%, 70.25%, and 68.78% amino acid sequence similarities between DasR, NagB, NagK, and NagA in *S. lincolnensis* and their homologs in *S. verticillus*, respectively. Based on these findings, as well as the prediction results from MEME and NCBI’s analysis of the whole genome sequence of wild-type *S. lincolnensis* NBRC_13054, it is speculated that the arrangement of these genes of *S. lincolnensis* used in this study is the same as that of *S. coelicolor* and *S. verticillus*. Subsequently, EMSA was conducted to validate the binding affinity of DasR towards these target genes, and the corresponding results are presented in Fig. 6. Although MEME predicted that there were *dre* sequences in the upstream regions of *lmrA*, *lmbR*, and *lmbU* of the three genes in the lincomycin biosynthesis gene, the EMSA results found that DasR did not bind to these locations. Combined with the transcriptome results, it could be inferred that the upregulation of the in-cluster gene transcription by DasR might be carried out through other indirect regulatory modes. The EMSA results for *dasA*, *nagB*, and *nagK* genes indicated a direct regulation mode to GlcNAc metabolism by DasR. As a regulatory protein, DasR also binds to the promoter region of its own gene *dasR* to exert negative feedback on its own transcription, and this auto-regulatory mechanism has been previously reported in literature (Xu et al. 2020). Additionally, in vitro assays were conducted to evaluate the activity of several negative regulators of lincomycin production, namely SLINC_3938, SLINC_4481, SLINC_4906, and SLINC_6156. This investigation was prompted by the observation that DasR exhibited binding affinity towards certain sites in *S. coelicolor* that lacked obvious similarity to *dre* (Swiatek-Polatynska et al. 2015). However, no direct binding was observed in our experiment.

## Discussion

As a common global regulator of GntR family, DasR, has been found to affect the growth, antibiotic synthesis, and other metabolic processes in *S. coelicolor* (Engel et al. 2020) and *Saccharopolyspora erythraea* (You et al. 2018) in recent years. The *DdasR* knockout strain and *OdasR* overexpressed strain were initially constructed in this study, followed by a comparison of lincomycin production and growth phenotypes between the engineered strains and the wild-type bacteria. Additionally, the effects of GlcNAc on the growth and fermentation of all three strains were investigated.

It was found that the *dasR* gene did not affect the spore production, but the wild type produced spores faster, and the two engineered strains produced spores with a slight delay. The spores produced by *OdasR* were slightly light gray, unlike the other two strains, which were pure white. The overexpression strain *OdasR* showed a 7.97-fold increase in lincomycin yield over the wild type. In *S. coelicolor*, the *dasR*-deficient BAP29 rarely produced spores on SFM solid medium, with a spore count about three orders of magnitude lower than that of the parental M145 and did not produce spores on R2YE medium (Rigali et al. 2008). In *S. roseosporus*, the effect of DasR on its morphology contrasted with *S. griseus* and *S. coelicolor*, where the *dasR*-deficient bacteria not only produced white spores but also spore formation at an earlier time, whereas the spore formation time of backfilled and *dasR* overexpressing strains did not differ significantly from that of the wild type (Chen et al. 2022). In addition, the pigment production of the *dasR* deletion strain was much lower than that of the wild type, but the difference between the back-complemented and overexpression strains was not significant, which also proved that DasR was associated with pigment production. Comparing the changes in lincomycin production, it is intuitively obvious that knockdown of the *dasR* gene significantly impairs the ability of the strain to produce lincomycin, which is contrary to the negative regulatory effects of most of the *dasR* genes. In rare cases, DasR positively regulates the production of antibiotics, such as the monensin production in *S. cinnamonensis* (Zhang et al. 2016). Overexpression, knockdown, and antisense repression of the *dasR* gene in *S. cinnamonensis* revealed that DasR binds to the promoter regions of the *monE*, *monT*, *monAIX*, and *monRII* genes within the monensin BGCs, upregulates their transcription, and facilitates the production of monensin, but the regulatory effects are related to environmental factors.

The DCW of the knockout strain *DdasR* was comparable to that of the wild-type strain, while the lincomycin production exhibited a decrease. Reduced and elevated expression of the *lmb* gene was directly responsible for the significant reduction and elevation of lincomycin production in *DdasR* and *OdasR*, and it was experimentally found that overexpression of the *lmbB1* gene effectively increased the production of lincomycin A, inhibited melanin production, and reduced the production of the by-product lincomycin B (Yang et al. 2020). Combined with the results of transcriptome analysis, most of the gene expression levels were downregulated after *dasR* knockdown, but most of the genes of the central carbon metabolism pathway were upregulated, and the number of differentially expressed genes decreased dramatically with the prolongation of the fermentation time, and the number of upregulated genes and downregulated genes gradually converged to the same number, which may imply that *dasR* regulation is closely related to the nutrient metabolism, especially the metabolism of the carbon source. As early as 2015, studies showed that the binding of DasR and *dre* targets in *S. coelicolor* was time-specific, such as the binding of DasR with primary metabolism-related genes during the vegetative growth phase (Swiatek-Polatynska et al. 2015). Considering the biomass of *DdasR* and WT at 48 h in this study, it is speculated that *S. lincolnensis* was still in the vegetative growth stage at this time, and DasR might have a negative regulatory relationship with these genes, showing a time-specific effect on these genes in the later stage. In addition, the upregulation of key genes related to central carbon metabolism after *dasR* gene knockout in *S. lincolnensis* was similar to the *dasR* deletion mutant of *S. cerulean* which upregulated the glycolysis, TCA cycle, gluconeogenesis and pyruvate metabolism, as well as the biosynthesis pathway of amino acids, nucleotides, and fatty acids metabolism. However, knockdown of *dasR* resulted in a significant decrease in lincomycin production, and the expression levels of all relevant genes in the lincomycin biosynthesis pathway showed downregulation.

It has been found that in *Streptomyces azureus* DasR also binds to a number of sites that have no obvious similarity to *dre* (Swiatek-Polatynska et al. 2015), and similarly, the non-coding RNA *scr5239* was found to control carbon source metabolism in Streptomyces, whereas the expression of *scr5239* is directly regulated by the global regulator protein DasR (Engel et al. 2020). Therefore, amplification of the relevant promoter region is also necessary to verify this in wet experiments. Since in vitro experiments did not identify *dasR* binding sites in the lincomycin synthesis gene cluster, it is hypothesized that *dasR* may control the synthesis of lincomycin, or even other secondary metabolites, through a more complex regulatory network. Similar phenomena were also found in SLINC_1596 (renamed as LcbR1) (Wang et al. 2023), inspired by these cases, DasR may regulate lincomycin biosynthesis via unknown transporter substance(s) or *dasR* homologs. In *S. verticillus*, there is no *dre* sequence within the bleomycin biosynthesis gene cluster, and DasR, although it does not directly regulate bleomycin synthesis, acts as an inhibitor of bleomycin production through indirect regulation (Chen et al. 2020). When overexpressing *dasR*, the dry weight of the knockout bacteria grew rapidly on the first day of fermentation and dropped significantly from the second day. However, lincomycin production peaked the next day. The higher expression of lincomycin biosynthesis genes was also observed. It was speculated that the overexpression of *dasR* gene might exert a stronger transcriptional inhibitory effect on central carbon metabolism or primary metabolism, thereby promoting an earlier activation of secondary metabolism in *S. lincolnensis*. In subsequent studies, it might be more beneficial to the industrial production of lincomycin by regulating the expression intensity or the expression time sequence of *dasR* in order to balance the synthesis of the antibiotic and the growth of the bacterium.

The dry weight of all three strains increased at the end of fermentation due to the addition of GlcNAc, and it is hypothesized that it may be due to the fact that the exogenous GlcNAc provided *S. lincolnensis* with more carbon sources, which promoted basal metabolism such as cell growth and development. Such results are consistent with previous studies showing an increase in cell dry weight after the addition of GlcNAc to the medium of *S. tsukubaensis* and verifying the use of the transcriptome that the addition of GlcNAc stimulated the transcription of genes related to glycolysis, pyruvate metabolism, and fatty acid biosynthesis (Ordonez-Robles et al. 2018).

Meanwhile, it was found that the addition of GlcNAc significantly inhibited the spore formation of *S. lincolnensis*, and the inhibition effect became more and more significant with the increase of the addition amount, and the addition of 100 mM of GlcNAc caused *DdasR* to almost lose the ability of spore formation, suggesting that *dasR* gene may have an effect on the morphological differentiation of *S. lincolnensis*. GlcNAc is the basic building block of many important polysaccharides in biological cells and is also an important component of bacterial cell wall peptidoglycan (Saito et al. 2007). GlcNAc was found to act as a signaling molecule regulating morphological differentiation and antibiotic synthesis in cells of *S. coelicolor*, and its metabolites inhibit the DNA-binding ability of the transcription factor DasR, thereby promoting the synthesis of secondary metabolites (Tenconi et al. 2015). In this study, we found that the addition of GlcNAc significantly reduced lincomycin production and significantly attenuated the promotion of lincomycin synthesis by *dasR*, suggesting that in *S. lincolnensis*, intracellular GlcNAc levels are an important component in the regulatory network of the global transcription factor DasR. In vitro EMSA experiments revealed that DasR inhibits the transcription of *dasA, nagK*, and *nagB* genes associated with GlcNAc metabolism by directly binding at the *dre* sequence position in the promoter region of these genes, a self-feedback regulation that may play an important role in maintaining the balance between bacterial growth and secondary metabolism. Exogenous addition of GlcNAc inhibits sporulation and lincomycin production in *S. lincolnensis* under enriched nutrient conditions, and the inhibition of the growth of engineered *S. lincolnensis* sporulation is particularly more pronounced, suggesting that DasR is associated with *S. lincolnensis* nutrient sensing (Rigali et al. 2006, 2008; Van Wezel et al. 2009).

**Fig. 7:**
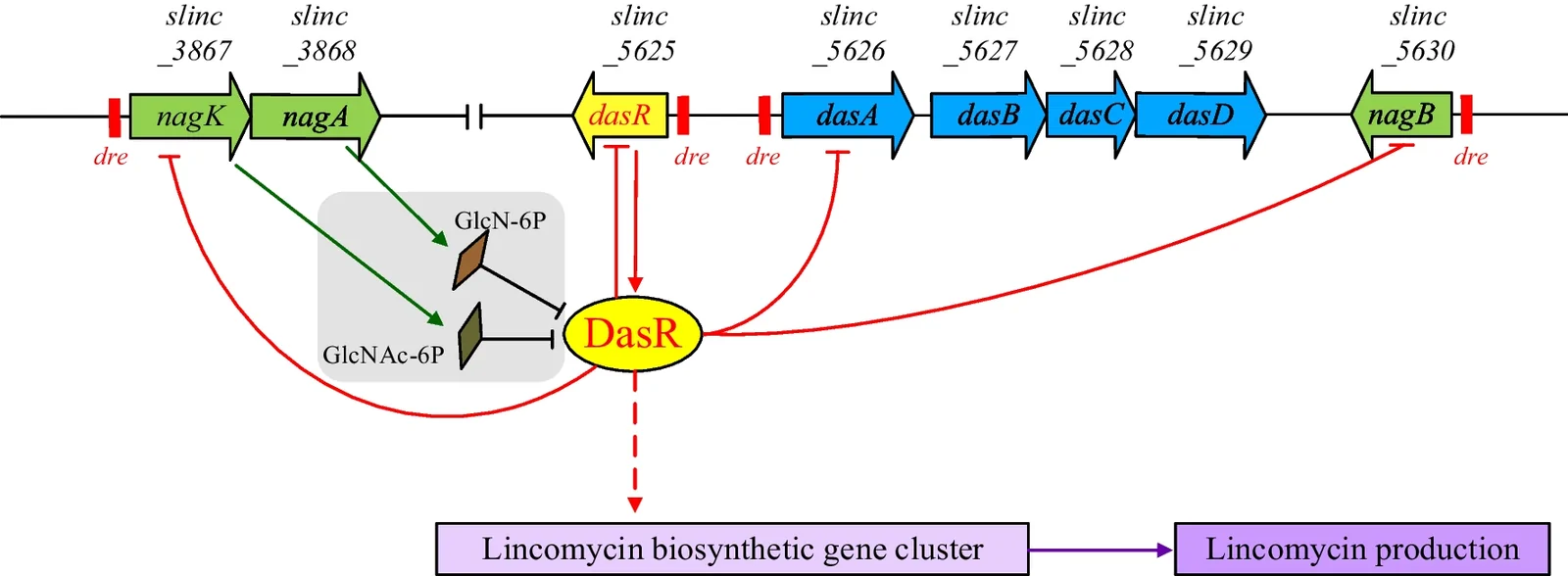
Regulation system of DasR involved in antibiotic production and GlcNAc metabolism. Pointed arrows represent activation. Flat-headed arrows represent repression. Solid lines represent direct regulation. Dashed line represents indirect regulation. Gray shadow: represents the speculation based on the references and the experimental results, and there is currently no direct data to confirm it

In conclusion (Fig. 7), DasR indirectly regulates lincomycin production by facilitating the intracellular transport of GlcNAc polymer through the ABC transporter system composed of DasABC. This process is catalyzed by DasD to convert GlcNAc polymer into GlcNAc monomer, which is further converted into GlcNAc-6P and GlcN-6P catalyzed by NagK and NagA enzymes (Nothaft et al. 2010). Both GlcNAc-6P and GlcN-6P specifically inhibit the binding of DasR to the promoter of the gene, releasing the original DNA target (Rigali et al. 2006), and then catalyzed by NagB to generate Fru-6P, which can enter the glycolytic pathway (Chen et al. 2019). The aforementioned process holds the potential to further optimize GlcNAc metabolism, thereby promoting mycelium vegetative growth while impeding antibiotic synthesis and spore formation. The exogenous addition of GlcNAc exerted inhibitory effects on sporulation and lincomycin production in *S. lincolnensis* under eutrophic conditions, thus proved this assumption. In addition, it has been reported that DasR also acts as a regulatory protein that binds to the promoter region of its own gene, *dasR*, and represses its own transcription (Xu et al. 2020). However, as a transcription suppressor, DasR exerts a positive influence on lincomycin production, suggesting its involvement in a complex regulatory mechanism that may encompass unidentified negative regulators of lincomycin biosynthesis. Therefore, many efforts should be down to excavate the hidden floor between DasR and lincomycin production.

## Supplementary Information

Below is the link to the electronic supplementary material.

Supplementary Material 1 (PDF 887 KB)

## Data Availability

The datasets generated and analyzed during the current study are available from the corresponding author on reasonable request.
