# Supplementary material for: Effects of the pleiotropic regulator DasR on lincomycin production in Streptomyces lincolnensis
Source: Appl Microbiol Biotechnol. 2024 Jun 15;108(1):373. doi: 10.1007/s00253-024-13201-7 (PMC11180011; doi:10.1007/s00253-024-13201-7)
Supplement: Supplementary file 1 — Supplementary Material 1 (PDF 887 KB) [file 253_2024_13201_MOESM1_ESM.pdf]

Applied Microbiology and Biotechnology

Supplementary Information

Effects of the pleiotropic regulator DasR on lincomycin production in *Streptomyces lincolnensis.*

Huihui Pai[1, #], Yiying Liu[1, #], Chuanbo Zhang[1.2,3], Jianyu Su[4,5*], Wenyu Lu[1,2,3*]

[1]School of Chemical Engineering and Technology, Tianjin University, Tianjin, PR China.

[2]Frontiers Science Center for Synthetic Biology, Tianjin University, Tianjin, PR China.

[3]Key Laboratory of System Bioengineering (Tianjin University), Ministry of Education, Tianjin, PR China.

[4]Key Laboratory of the Ministry of Education for Conservation and Utilization of Special Biological Resources in the Western, Yinchuan 750021, China.

[5]College of Life Science, Ningxia University, Yinchuan 750021, Ningxia, China.

*Corresponding author: Jianyu Su, Email: su_jy@nxu.edu.cn

*Corresponding author: Wenyu Lu, Email: wenyulu@tju.edu.cn

# Huihui Pai and Yiying Liu contribute equally to this article.

Content

Table S1-Primers used to construct and check pKCcas9dO-dasR 3
Table S2-Primers used to construct and check pIB139-dasR 4
Table S3-Primers for quantitative real-time PCR 4
Table S4-First PCR primers for the promoter regions of the target genes 5
Fig. S1-Construction of *dasR* knockout vector and strain. 7
Fig. S2-Construction of dasR overexpressing plasmid pIB139-*dasR*. 8
Fig. S3-Construction of DasR protein purification plasmid pCold II-*dasR*. 9
Fig. S4-Analysis of gene differential expression in *S. lincolnensis* at different periods. 9
Fig. S5-Sequence of dre predicted by MEME of *S. lincolnensis* 10

Table S1-Primers used to construct and check pKCcas9dO-dasR

Primer	Sequence（5’-3’）
P-dasRgRNA-F	AGTGTCAGCAGTGCGGAGAACGAGTTTTAGAGCTAGAAATAGCAAGTTAAAATAAGGC
dasRgRNA-P-R	AACTCGTTCTCCGCACTGCTGACACTAGTATTATACCTAGGACTGAGCTA
gRNA-dasRdown-F	GAGTCGGTGCTTTTTTTGAGAGGCGATGAAGCCGTACGC
dasRdown-gRNA-R	TACGGCTTCATCGCCTCTCAAAAAAAGCACCGACTC
dasRdown-dasRup-F	CGCCCCTTGGGTGTGCCGCTCTGCCTTCCCGGCTCCG
dasRup-dasRdown-R	GCCGGGAAGGCAGAGCGGCACACCCAAGGGGCGCGG
dasRup-P-F	ACCTCTCCTCGCCCGGGTCGAAGCTTGGCACTGGCCGT
P-dasRup-R	TGCCAAGCTTCGACCCGGGCGAGGAGAGGTTGCCCTTC
P2-F	GCTGATGGAGCTGCACATGAACCAAAAGGATCTAGGTGAAG
P1-R	TTCATGTGCAGCTCCATCAGCAAAAGGGGATGATAAGTTTAT
check-dasRdown-F	GATCATCGTCGGCGTCATCTTC
check-dasRdown-R	ACTTCTTCGACGGGACCTTGT
check-dasRup-F	TAGCTGCGCCGATGGTTTCTAC
check-dasRup-R	ACAAGAACGCCAAGCTCGAC
check-P-F	AGGCGGTGCTACAGAGTTCTT
check-P-R	AGCGTCGTGTTGGCATCGTGT
DdasR-F	ACTTGCCCTGGCCGTAGACGATG
DdasR-R	AGAGCAGGATGTGCGGTTGCG

Table S2-Primers used to construct and check pIB139-dasR

Primer	Sequence（5'-3'）
139-dasR-F	TAGGATCCACATATGATGAGCACCGACGTCAGCAGT
139-dasR-R	CATGATTACGAATTCTCAGTCCACGGGCCTTTTCAAGCG
dasR-139-F	GGCCCGTGGACTGAGAATTCGTAATCATGTCATAGCTG
dasR-139-R	GACGTCGGTGCTCATCATATGTGGATCCTACCAACCGG
check-139-F	ACGGAAGACGTAGCGGCGTAG
check-139-R	TCCGGCTCGTATGTTGTGTG
check-139-2F	TTCGACACCTCGCGCACCAC
check-139-2R	TGCCCTTGCCCTGGATGCGC
OdasR-F	ATGCAGCTCTCGGAGGGCGAAG
OdasR-R	TCAGTCCACGGGCCTTTTCAAGCG

Table S3-Primers for quantitative real-time PCR

Primer	Sequence（5'-3'）
q-16s-F	GCAGCGACGATGAACCACT
q-16s-R	GGTATCCGGTATTAGACCCC
q-lmrA-F	ATGTCTGTCTTCGCTCGTGC
q-lmrA-R	TGGAGCACGGCGAGGAAG
q-lmbB1-F	ACCATGTCGGTGTCCAGACC
q-lmbB1-R	AAGCCGTGAGTGGGTGAGT
q-lmbC-F	CGTCCTCCGTTCGACTCTCC
q-lmbC-R	AGCTCCCCATAGGTGACCG
q-lmbD-F	ACGTCGAAGAGGTCACAGCG
q-lmbD-R	ACCGACCCTCTTCGCCAC
q-lmbF-F	ACGACGGCTGGCTGATCC
q-lmbF-R	GACGTGCAGCAGGTACAGC
q-lmbN-F	ACGAGACCGAACTCACCGC
q-lmbN-R	AGTTCCATGGGTGGGATGGG
q-lmbR-F	CTTTCTGGATACGCCGGACC
q-lmbR-R	TGCCCGTCCAGTCCTCGATT
q-lmbV-F	ACCTCTGACGCCCTTCCG

q-lmbV-R	AGGTGTCTTCCCTCGTCGG
q-lmrB-F	ACCCCGACATCATCAAGCTG
q-lmrB-R	AGCTCAACCGCGGTGACA
q-lmbY-F	GTCGTGATCCTGCCCGAGC
q-lmbY-R	CTCAGCCAGCGCCACTTCAC
q-lmrC-F	ACGCGAGTATTGTCTGCACC
q-lmrC-R	AGTGTGGACTTTCCCGCGC
q-nagK-F	GTGAGACATGTCATCGCCCT
q-nagK-R	ATGCCCTCGACCACGGCG
q-dasR-F	ATGAGCACCGACGTCAGC
q-dasR-R	TGCGTCTCCGTCATGTCG
q-dasA-F	ATCGGTATCGCGGGCATG
q-dasA-R	CTGCGCGTCGACCGTGAG
q-nagB-F	GTGGAAGTTGTCATCGTTC
q-nagB-R	TCGAGCCGGTCGCCACAC

Table S4-First PCR primers for the promoter regions of the target genes

Primer	Sequence（5’-3’）
E-lmrA-F	AGCCAGTGGCGATAAGATATCCTCTTTCCCCGGGAG
E-lmrA-R	AGCCAGTGGCGATAAGTCCATCCGGCCCGTAGCGTC
E-lmbR-F	AGCCAGTGGCGATAAGATCGAGGACCTGACCGGCAC
E-lmbR-R	AGCCAGTGGCGATAAGCAGCTCCACCACGAGGTTGG
E-lmbU-F	AGCCAGTGGCGATAAGAACAAGGCTTCGTCGGGGGT
E-lmbU-R	AGCCAGTGGCGATAAGATCCCGAGGTCGACGCCCGA
E-nagK-F	AGCCAGTGGCGATAAGTCCTACGCCGCCGCACTGCC
E-nagK-R	AGCCAGTGGCGATAAGATGACATGTCTCACGGGGGAC
E-dasR-F	AGCCAGTGGCGATAAGCGAGCTACATATGCAGGAACC
E-dasA-F	AGCCAGTGGCGATAAGCCTCCTCAGGGATGCCCACA
E-dasA-R	AGCCAGTGGCGATAAGGGTTCCTGCATATGTAGCTCG
E-nagB-F	AGCCAGTGGCGATAAGTTCGCTGACGTAGGGACCGC
E-nagB-R	AGCCAGTGGCGATAAGGCTGGGCCTGCCGTTCTCAA
E-3938-F	AGCCAGTGGCGATAAGTCACTCGGTCTTCCCGTCCG
E-3938-R	AGCCAGTGGCGATAAGCACGCAGCCCGATCTCAGCCAT

E-4481-F	AGCCAGTGGCGATAAGATCTCGAACTCCACCAGCGTC
E-4481-R	AGCCAGTGGCGATAAGACGCACGTACGCAACTCCTTTC
E-4906-F	AGCCAGTGGCGATAAGAGGACACAGGGCAGTAGGGC
E-4906-R	AGCCAGTGGCGATAAGAGGGCCTGGATGAGCTGCCT
E-6156-F	AGCCAGTGGCGATAAGAGCTCTAGCAACAGACTTCGC
E-6156-R	AGCCAGTGGCGATAAGATGACTCAAAAGACTCTCCTC

Fig. S1-Construction of *dasR* knockout vector and strain.

a Construction of *dasR* disruption plasmid pKCcas9dO-*dasR*. **b** *DdasR* PCR and sequencing identification. Line 1 and 2 indicated *DdasR*. Line 3 indicated NBRC_13054. **c** The arrow indicated the *dasR* knock-out position.

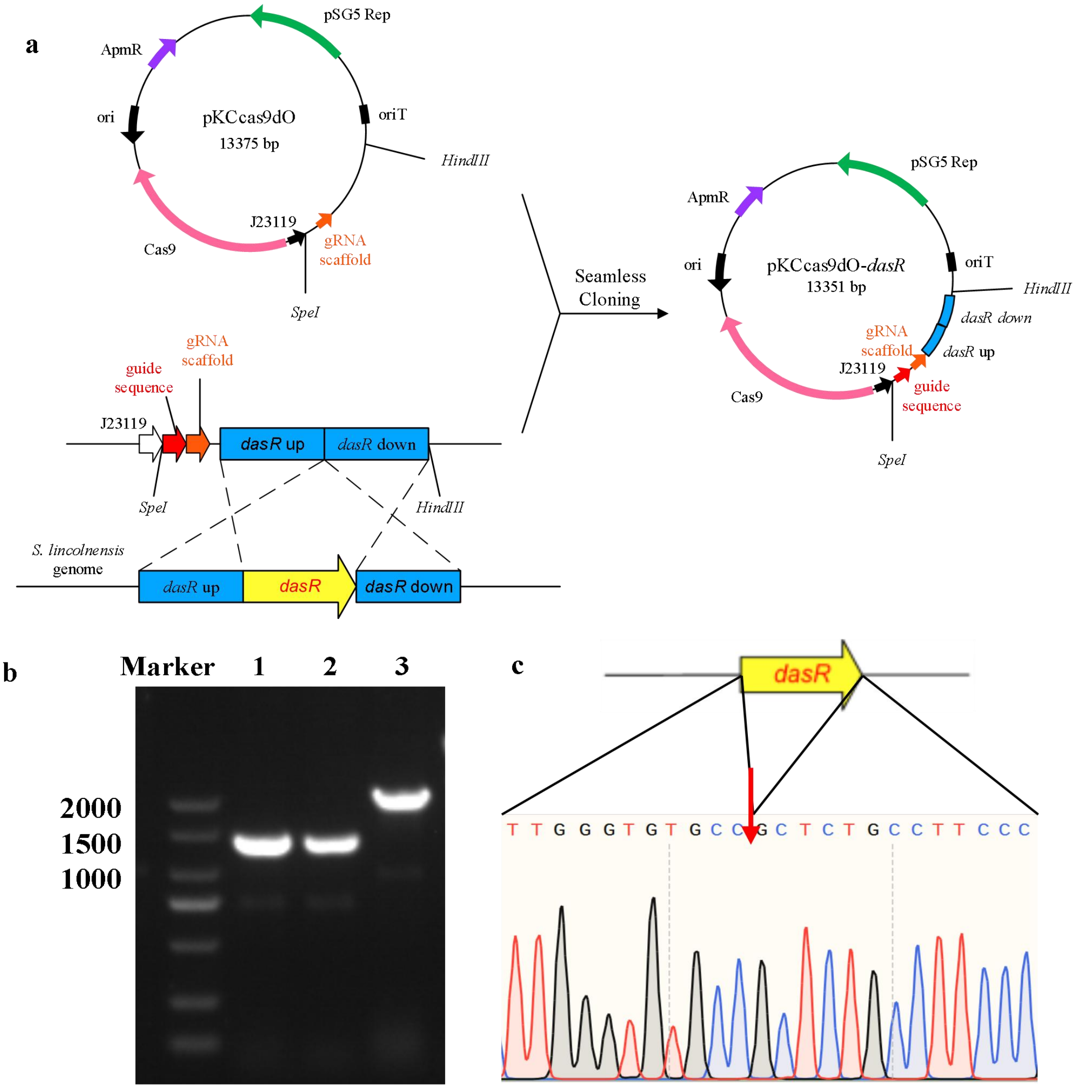

Fig. S2-Construction of dasR overexpressing plasmid pIB139-*dasR*.

a Construction of *dasR* overexpressing plasmid pIB139-*dasR*. **b** *OdasR* PCR and sequencing identification. Line 1 indicated NBRC_13054. Line 2 indicated pIB139-*dasR* plasmid. Line 3 indicated *OdasR*. **c** The arrow indicated the *dasR* knock-out position.

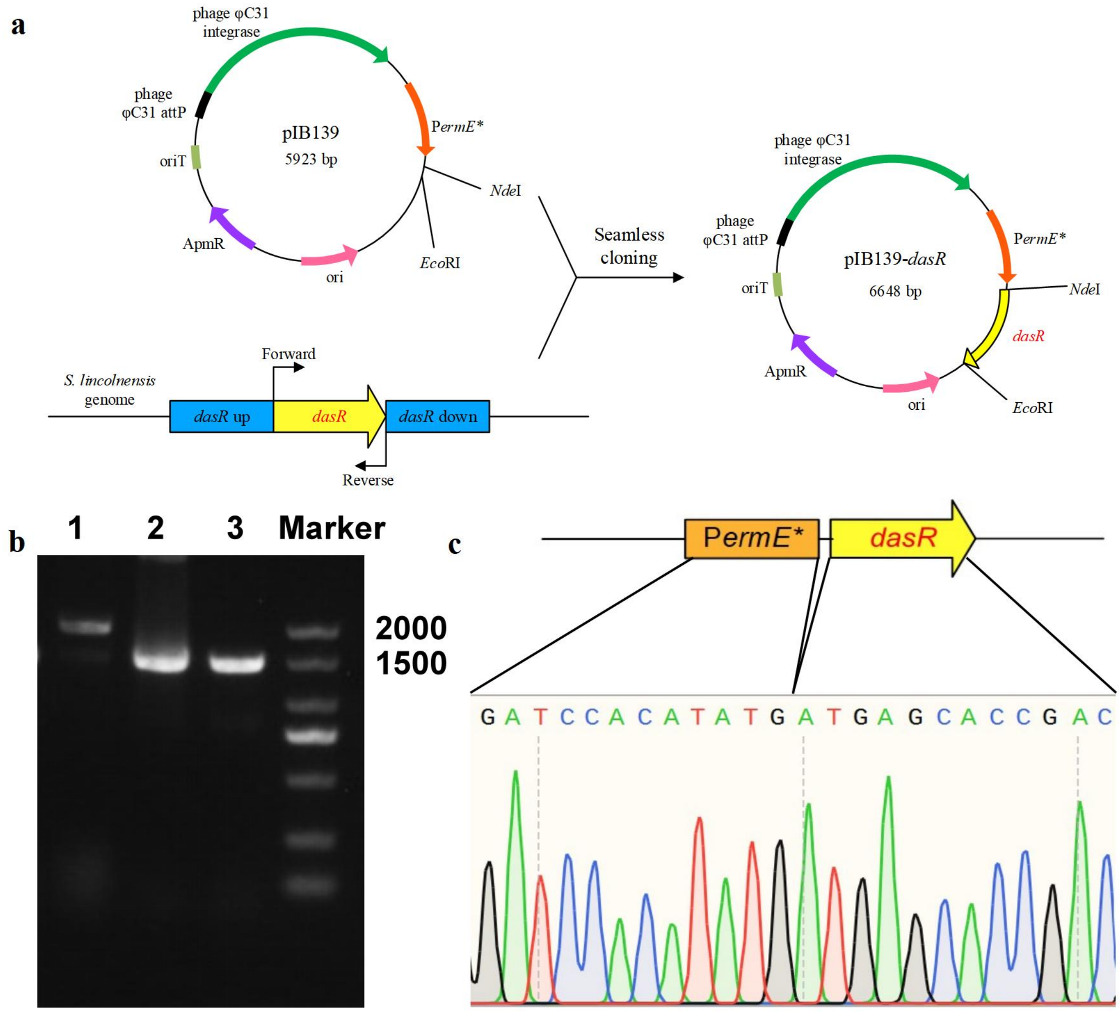

Fig. S3-Construction of DasR protein purification plasmid pCold II-*dasR*.

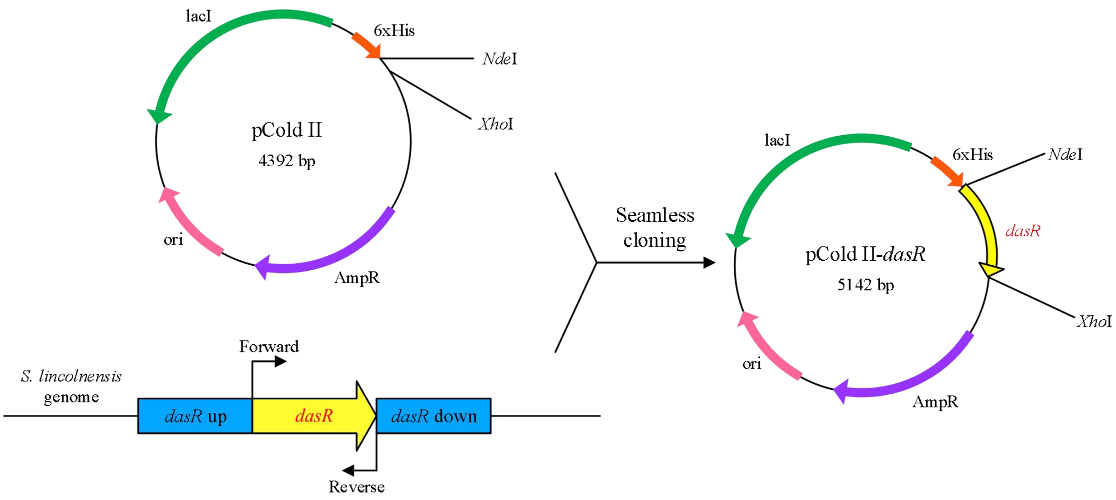

Fig. S4-Analysis of gene differential expression in *S. lincolnensis* at different periods.

a WT VS *DdasR* differentially expressed genes in *lmb*. **b** Differential expression of *nagK*, *nagA* and *nagB* in WT VS *DdasR* at three stages.

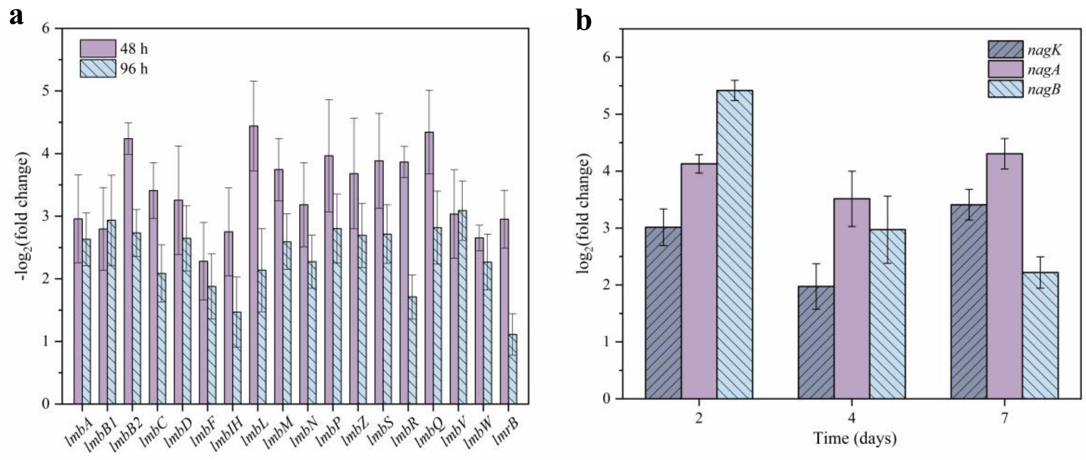

Fig. S5-Sequence of dre predicted by MEME of *S. lincolnensis*

Red letters indicate bases where the predicted sequence differs from the *dre* sequence.

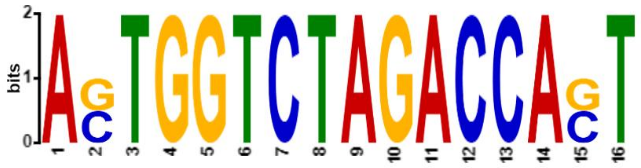

dre **sequence**	**A**	**G/C**	**T**	**G**	**G**	**T**	**C**	**T**	**A**	**G**	**A**	**C**	**C**	**A**	**G/C**	**T**
lmrA	C	G	T	G	G	T	C	G	A	G	A	T	C	G	G	T
lmbR	A	C	T	G	G	C	C	T	C	G	T	C	C	A	G	C
lmbU	C	C	T	G	G	G	G	G	A	G	A	C	C	A	C	C
dasR	G	G	T	G	G	A	C	T	A	G	A	C	C	A	G	A
dasA	A	A	T	G	G	T	G	T	A	G	A	C	C	A	G	T
nagB	A	T	T	G	G	T	C	T	A	A	A	C	C	A	C	A
nagK	A	G	T	G	G	A	C	T	A	G	A	C	C	T	C	T
